# Electrokinetic Potential of Basic Zinc Sulfate and of Products of Its Ion Exchange

**DOI:** 10.3390/molecules31071112

**Published:** 2026-03-27

**Authors:** Sebastian Skupiński, Marta Kalbarczyk, Daniel Kamiński, Marek Kosmulski

**Affiliations:** 1 Laboratory of Electrochemistry, Department of Electrical Engineering and Smart Technologies, Lublin University of Technology, Nadbystrzycka 38, 20-618 Lublin, Poland; s.skupinski@pollub.pl (S.S.); m.kalbarczyk@pollub.pl (M.K.); 2 Department of Organic Chemistry and Crystallography, Institute of Chemical Sciences, Faculty of Chemistry, Maria-Curie-Skłodowska University in Lublin, Pl. M Curie-Skłodowskiej 3, 20-031 Lublin, Poland; daniel.kaminski@mail.umcs.pl

**Keywords:** zinc basic salts, electrophoresis, TGA, XRD, specific surface area

## Abstract

Basic zinc sulfate with an empirical formula of ZnSO_4_∙3 Zn(OH)_2_∙3.5 H_2_O (or Zn_4_SO_4_(OH)_6_∙3.5 H_2_O) was precipitated using stoichiometric amounts of ZnSO_4_ and NaOH, followed by drying and storage in air. The XRD pattern suggests that the product contains tri- and tetrahydrate of basic zinc sulfate. Penta-, mono-, and hemihydrates of basic zinc sulfate can be produced by storing the original material in air at various temperatures and humidity levels, and especially by immersion in aqueous solutions. The precipitate was characterized by its specific surface area and zeta potential, and it has an isoelectric point (IEP) at pH 8.9. Ion exchange with an excess of CuSO_4_ results in conversion to brochantite Cu_4_(OH)_6_SO_4_ (as detected by XRD) and in an increase in the specific surface area. The conversion was complete at room temperature with a sufficient excess of CuSO_4_, but it was not complete at 50 or 60 °C. Apparently, the conversion into brochantite is exothermic. The IEP of brochantites obtained from ZnSO_4_∙3 Zn(OH)_2_∙3.5 H_2_O by ion exchange was at a pH of about 10, which is higher than the previously reported IEP.

## 1. Introduction

Rechargeable, mildly acidic zinc ion batteries are considered an alternative to other types of batteries for large-scale energy storage systems. Zinc-ion batteries have a moderate energy density, but they are more cost-efficient, environmentally friendly, and safer than other types of batteries. Poor cycling stability is among its most serious challenges, and it has to be managed to make mildly acidic zinc-ion batteries more competitive for energy storage systems.

ZnSO_4_ is a typical electrolyte in mildly acidic zinc-ion batteries. Its pH varies in both anode and cathode compartments during charging–discharging cycles; that is, the electrolyte is alternately acidified and basified. This leads to precipitation of zinc hydroxy sulfate according to the following reaction:

4 ZnSO_4_ + 6 OH^−^ → Zn_4_(OH)_6_SO_4_ + 3 SO_4_^2−^. (1)

This reaction is partially reversible; that is, zinc hydroxy sulfate is digested (dissolved) on the next charging–discharging stage:

Zn_4_(OH)_6_SO_4_ + 6 H^+^ → ZnSO_4_ + 3 Zn^2+^ + 6 H_2_O, (2)

However, its reversibility is only partial. The growth of Zn_4_(OH)_6_SO_4_ crystals impairs the performance of the batteries and results in their premature degradation [1]. A solution for the “zinc hydroxy sulfate problem” in zinc batteries may substantially improve their cycling stability. More discussions of this problem can be found in [2] and in the references therein.

Zinc hydroxy sulfates have been studied long before mildly acidic zinc-ion batteries were developed. Zn_4_(OH)_6_SO_4_ is the most well-known zinc hydroxy sulfate, and its formula is analogous to brochantite Cu_4_(OH)_6_SO_4_, which is a hydroxy sulfate of copper, but unlike anhydrous brochantite, Zn_4_(OH)_6_SO_4_ occurs in the form of hydrates. A mixed Zn-Cu hydroxy sulfate, namuwite, (Zn,Cu)_4_(OH)_6_SO_4_∙4 H_2_O, is also hydrated. The isomorphic substitution of Zn by Cu in namuwite and in other zinc hydroxy sulfates has been extensively studied [3]. With low Cu-to-Zn ratios, the mixed Zn-Cu basic sulfate crystallizes as a 4-hydrate, but a mixture of Cu-substituted namuwite and brochantite is obtained with higher Cu-to-Zn ratios. This result shows that even very similar ionic radii are not sufficient to allow isomorphic substitutions of Zn with Cu in their basic sulfates over a broad range of Cu-to-Zn ratios. Crystallization is affected by the Jahn–Teller effect, which is substantial with Cu and negligible with Zn. Namuwite allows replacement of the highest fraction of Zn with Cu among the (Zn,Cu)_4_(OH)_6_SO_4_∙x H_2_O minerals.

The following hydrates of Zn_4_(OH)_6_SO_4_ have well-established XRD patterns: pentahydrate (osakaite), tetrahydrate (namuwite), trihydrate (lahnsteinite), monohydrate, and hemihydrate. The XRD pattern of anhydrous salt is very similar to that of hemihydrate. On top of these well-defined compounds, several studies report on XRD patterns of the hydrates of Zn_4_(OH)_6_SO_4_, which do not match any of the above patterns.

Moezzi et al. [4] precipitated Zn_4_(OH)_6_SO_4_∙4 H_2_O from ZnSO_4_ and NaOH at room temperature using a slight excess of ZnSO_4_. The powder was dried for 30 min. At 60 °C, it showed an XRD pattern typical of a tetrahydrate. The XRD patterns of the original tetrahydrate heated at various temperatures are also reported. The following correlation was found between x, the number of water molecules in Zn_4_(OH)_6_SO_4_∙x H_2_O, and y, the position of the peak for interlayer spacing (as 2θ for Cu Kα) [4]:

y = 0.0361 x^2^ − 1.1513 x + 12.785. (3)

Wang et al. [5] dried their synthetic Zn_4_(OH)_6_SO_4_ at various temperatures. Drying at 25 °C resulted in Zn_4_(OH)_6_SO_4_∙5 H_2_O. Drying at 150 °C resulted in Zn_4_(OH)_6_SO_4_∙0.5 H_2_O (both formulas confirmed by XRD). Drying at 70 °C resulted in a product with the largest peak corresponding to a d-spacing of 0.855 nm. Such a peak is not found in any of the well-known XRD patterns of Zn_4_(OH)_6_SO_4_ hydrates. The authors argue that this peak is halfway between the main peaks of the XRD patterns of tri- and monohydrates. This argument is based on the aforementioned correlation (Equation (3)). According to Equation (3), the d-spacing of 0.855 nm corresponds to 2.3 water molecules per one Zn_4_(OH)_6_SO_4_ unit, while the main peaks in the penta- and hemihydrate presented in [5] correspond to 4.6 and 0.4 water molecules per one Zn_4_(OH)_6_SO_4_ unit, respectively.

Guner et al. [6] dried their synthetic Zn_4_(OH)_6_SO_4_ at 200 °C. The original powder was a trihydrate, and the dried powder was a hemihydrate, as confirmed by XRD. No unexplained peaks were found in these materials.

Delcheva et al. [3] studied precipitates obtained from ZnSO_4_ and NaOH at different proportions of these reagents. The tetrahydrate was precipitated at pH 6.5–7, while the trihydrate with an admixture of Zn(OH)_2_ was precipitated at pH above 12.

Stanimirova et al. [7] obtained their pentahydrate from ZnO and ZnSO_4_ solution. The powder was dried in humid air to avoid an excessive loss of water. Then, the pentahydrate was dried using different methods (temperature and zeolite) and rehydrated. This resulted in powders with XRD patterns of lower hydrates (down to hemihydrate) on drying, and they exhibited XRD patterns of higher hydrates (up to pentahydrate) on rehydration. No product other than the aforementioned well-defined hydrates was observed. Only short ranges of the XRD patterns were measured to avoid conversion of the studied materials into another hydrate during the measurement, because the possibility of controlling the temperature and humidity during XRD measurements is limited.

Partial transformation of ZnO into basic sulfate on immersion in dilute ZnSO_4_ solution was studied by Dong et al. [8]. The XRD patterns of the resulting materials showed peaks characteristic for Zn oxide and hydrous hydroxide, and additional peaks were attributed to Zn_4_(OH)_6_SO_4_∙5 H_2_O, which grew with the time of immersion at the expense of the ZnO peaks. With longer immersion times, an additional peak appeared, corresponding to a d-spacing of 0.924 nm, which is close to 0.929 nm, the interlayer spacing in Zn_4_(OH)_6_SO_4_∙3 H_2_O.

Xue et al. [9] obtained their hemihydrate from NaOH and ZnSO_4_ solutions at 150 °C. The precipitate was washed and dried at 60 °C. Two specimens obtained at different NaOH-to-ZnSO_4_ ratios showed XRD patterns characteristic for hemihydrate.

Delcheva et al. [10] report on a 2.5 hydrate of Zn_4_(OH)_6_SO_4_ with an interlayer distance of 1.784 nm, obtained from basic Zn nitrate and Na_2_SO_4_. Their result is not consistent with Equation (3). A similar product, described as the 2–2.25 hydrate of Zn_4_(OH)_6_SO_4_, was obtained by Stanimirova et al. [11,12] by aging of namuwite with 1 M NaI followed by washing and drying. Their material also showed a line corresponding to an interlayer distance of about 1.78 nm, and different explanations of the origin of such a line have been discussed. The hydration level in the “new phase” reported by Stanimirova et al. is similar to that postulated in the aforementioned paper by Wang et al.

Zn_4_(OH)_6_SO_4_ and its hydrates are not the only well-defined basic zinc sulfates. Germann et al. [13] obtained 5 Zn(OH)_2_∙2 ZnSO_4_ (anhydrous) by heating an aqueous solution composed of ZnSO_4_, boric acid, and borax at 120 °C, followed by drying of the precipitate in a vacuum. No details on the possible hydration of this product in wet air are reported.

The above examples show that hydration and dehydration of Zn_4_(OH)_6_SO_4_ have a substantial effect on the XRD pattern, and this phenomenon is especially important in the studies of ion-exchange reactions involving Zn_4_(OH)_6_SO_4_ and its hydrates. Namely, the changes in the XRD pattern caused by immersion of Zn_4_(OH)_6_SO_4_∙x H_2_O in aqueous solutions followed by washing and drying may be due not only to the transformation of Zn_4_(OH)_6_SO_4_ into another compound but also to the changes in its hydration level. In other words, a change in the XRD pattern of the original material on attempted ion exchange does not prove that Zn_4_(OH)_6_SO_4_ was transformed into another compound. In this respect, Zn_4_(OH)_6_SO_4_ is very different from brochantite and paratacamite [14], which are anhydrous, and unsuccessful ion-exchange results in an unchanged XRD pattern in these Cu minerals. The hydration of Zn_4_(OH)_6_SO_4_ is relevant to the present study, but a detailed investigation of the hydration of Zn_4_(OH)_6_SO_4_ was not the goal of this study.

The aforementioned hydration and dehydration make ion-exchange studies involving Zn_4_(OH)_6_SO_4_ more difficult than with other materials, and only a few researchers undertook this task. Stanimirova [15] studied the reactions of namuwite (tetrahydrate) with chlorides of the I and II group metals. The reactions with LiCl and MgCl_2_ resulted in simonkolleite Zn_5_(OH)_8_Cl_2_∙H_2_O. The reactions with NaCl, KCl, and CaCl_2_ resulted in gordaite NaZn_4_SO_4_(OH)_6_Cl∙6 H_2_O and its K- and Ca-analogs. The reactions with SrCl_2_ and BaCl_2_ resulted in mixtures of corresponding gordaites and metal sulfates. Delcheva et al. [10] studied reactions of their Zn_4_(OH)_6_SO_4_∙2.5 H_2_O with chlorides of the I and II group metals. The reaction with NaCl resulted in a mixture of namuwite, gordaite, and the initial material. The reaction with CaCl_2_ resulted in a mixture of Ca-gordaite and the initial material. The reaction with 1 M SrCl_2_ resulted in a mixture of simonkolleite and SrSO_4_. The reaction with 0.5 M SrCl_2_ resulted in a mixture of Sr-gordaite, simonkolleite, SrSO_4_, and the initial material.

Unlike zinc and copper basic sulfates, which have been extensively studied, the basic sulfates of other transition metals are less well-documented. It can be speculated that reactions analogous to (1), with ZnSO_4_ replaced by another transition metal sulfate, may produce basic sulfates of transition metals. Saarinen et al. [16] mixed a NiSO_4_ solution with understoichiometric amounts of NaOH at different conditions. Their precipitates contained only 1.8 and 1.2% of sulfur, respectively, and their XRD patterns matched that of Ni(OH)_2_, except that, in one precipitate, the reflection corresponding to a d-spacing of 0.4605 nm was split into two reflections corresponding to a d-spacing of about 0.42 and 0.52 nm, respectively. The present authors are not aware of analogous experiments with Co(II). Basic sulfates of cobalt were mentioned in several studies reporting on MOFs [17], and in the references therein, but the investigation of basic sulfates of cobalt was not the main goal of these studies. Moreover, Co(II) was partially oxidized to Co(III). A mixed Zn-Co basic sulfate has been studied [18]. The unsuccessful attempts to obtain basic sulfates of transition metals other than Zn or Cu do not imply that such compounds cannot be obtained. Cation exchange is among the potential methods to synthesize such compounds. Basic sulfates of Co and Ni are not directly related to the aforementioned rechargeable, mildly acidic zinc ion batteries. On the other hand, mixed electrolytes like ZnSO_4_ + MnSO_4_ have been proposed for those batteries. The Mn cations (and other cations) may partially replace Zn and thus affect the crystallization of Zn basic sulfates, their degree of hydration, etc. Therefore, fundamental research on cation exchange, also in systems that are not directly related to batteries, may lead to a better understanding of zinc basic sulfates and, thus, indirectly to a better understanding of rechargeable, mildly acidic zinc ion batteries.

The present authors are not aware of any studies of ion exchange involving Zn_4_(OH)_6_SO_4_∙x H_2_O and transition metal cations. The results of successful Zn-Cu exchange and of a few less successful experiments are presented in this study.

There are no reliable data on the solubility product or on other standard thermodynamic functions of Zn_4_(OH)_6_SO_4_∙x H_2_O in the literature. The level of hydration of Zn_4_(OH)_6_SO_4_ is very difficult to control, and these thermodynamic functions certainly depend on the level of hydration. Therefore, the direction of Zn-Cu exchange cannot be predicted/verified using chemical thermodynamics. We also emphasize that only the replacement of Zn by Cu was studied, and the replacement of Cu by Zn was not. In other words, the present results do not imply that brochantite cannot be converted into a corresponding Zn-salt with a sufficient excess of Zn ions in solution.

The electrokinetic properties of basic zinc sulfate at low pH may be relevant to mildly acidic zinc ion batteries; namely, the stability of colloidal dispersions against coagulation is governed by the ζ potential. This makes a difference if the basic zinc sulfate in the batteries is present as a stable colloidal dispersion or if it rapidly coagulates and settles down.

## 2. Results and Discussion

### 2.1. Original Particles

#### 2.1.1. XRD and the Empirical Formula of the Original Particles

**Figure 1:**
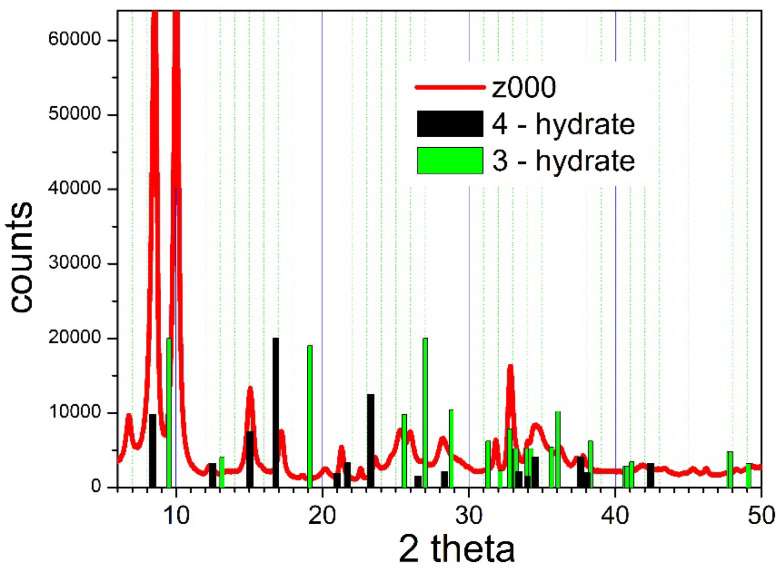
The XRD pattern of basic zinc sulfate (Z000).

The original particles (Z000) lost 37.4% of their mass on calcination at 1200 °C. Assuming that ZnO is the product of thermal decomposition, this figure corresponds to the following composition: Zn_4_(OH)_6_SO_4_∙3.5 H_2_O. Another specimen of Z000 was dissolved in dilute HCl, and BaSO_4_ was precipitated with excess of Ba(NO_3_)_2_. The mass of precipitated BaSO_4_ confirms a Zn:S molar ratio of 1:1 in Z000, with about 3.5 molecules of H_2_O per one Zn_4_(OH)_6_SO_4_ unit. The XRD pattern of Z000 is presented in Figure 1.

Figure 1 indicates that most major peaks of Z000 can be explained in terms of tetrahydrate. The major peak at 2θ of 8.515° (d-spacing of 1.038 nm) corresponds to 4.2 water molecules per Zn_4_(OH)_6_SO_4_ unit according to Equation (3), while the major peaks of the 4-hydrate found by others correspond to 4.2–4.4 water molecules per Zn_4_(OH)_6_SO_4_ unit according to Equation (3). The major peak at 2θ of 10.01° (d-spacing of 0.883 nm) corresponds to 2.6 water molecules per Zn_4_(OH)_6_SO_4_ unit according to Equation (3), and it only roughly matches a peak found by others for the trihydrate at a d-spacing of 0.93 nm. It is likely that Z000 is a mixture of tri- and tetrahydrates, in which the hydrates form separate crystallites. The absence of peaks at 19.126° (d-spacing of 0.464 nm) and 27.017° (d-spacing of 0.33 nm) in Z000, which are reported by others for trihydrates, is a weak point of this hypothesis. On the other hand, the XRD patterns of basic zinc sulfates are strongly affected by a preferential orientation of platelets (cf. Appendix B) in specimens prepared for XRD measurements, and very weak higher-order reflections are commonplace in these compounds. The XRD pattern of Z000, dominated by large peak(s) at 8–12°, confirms the layer structure of basic zinc sulfate, which was also emphasized by others.

The XRD pattern presented in Figure 1 is complicated due to the presence of multiple phases. Moreover, the proportions between particular phases are affected by the humidity level and temperature, and they may even change in the course of collecting the XRD pattern. Interestingly enough, an alternative explanation of the XRD pattern of Z001 can be offered when the “new phase” (2–2.25 hydrate) recently discovered by Stanimirova et al. [12] is taken into account on top of the other hydrates well-established in the literature. An extended analysis of Figure 1 is presented in the Appendix A.

#### 2.1.2. TGA

**Figure 2:**
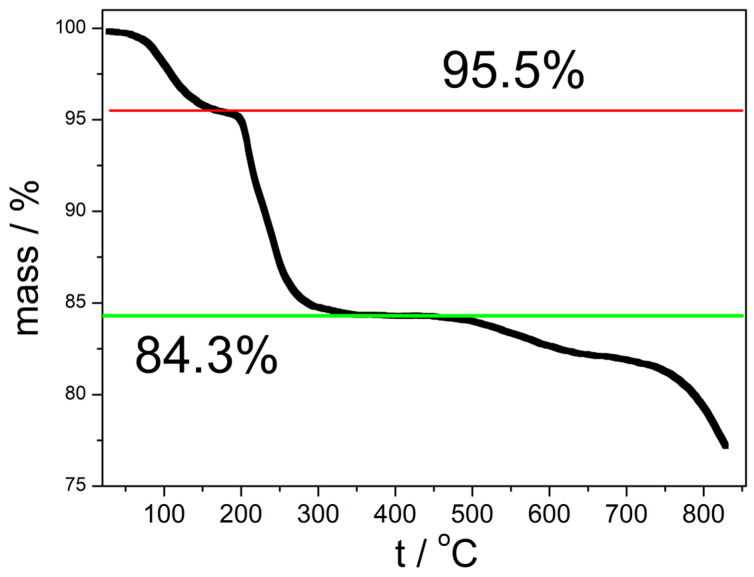
The TGA curve of Z0. The red line (95.5%) corresponds to the loss of 1.2 water molecules per one hypothetical Zn_4_(OH)_6_SO_4_∙1.2 H_2_O unit. The green line corresponds to the loss of 4.2 water molecules per one hypothetical Zn_4_(OH)_6_SO_4_∙1.2 H_2_O unit.

The thermogravimetric curve of the original material before its rehydration in air (Z0) is presented in Figure 2. The horizontal lines correspond to the complete loss of water of hydration (dehydration to anhydrous Zn_4_(OH)_6_SO_4_) and to the complete loss of both water of hydration and of constitution (dehydration to anhydrous ZnO and ZnSO_4_) in the hypothetical Zn_4_(OH)_6_SO_4_∙1.2 H_2_O. The TGA curve is complicated, and it does not show clear plateaus.

The DSC curve has two major endothermic peaks at 213.5 and 242.7 °C and numerous minor endothermic peaks. The thermogravimetric curve presented in Figure 2 is similar to analogous curves reported by others [4,6,7,9] for various hydrates of Zn_4_(OH)_6_SO_4_.

#### 2.1.3. SSA

The specific surface area of Z000 is 3.96 m^2^/g.

#### 2.1.4. Electrokinetics

**Figure 3:**
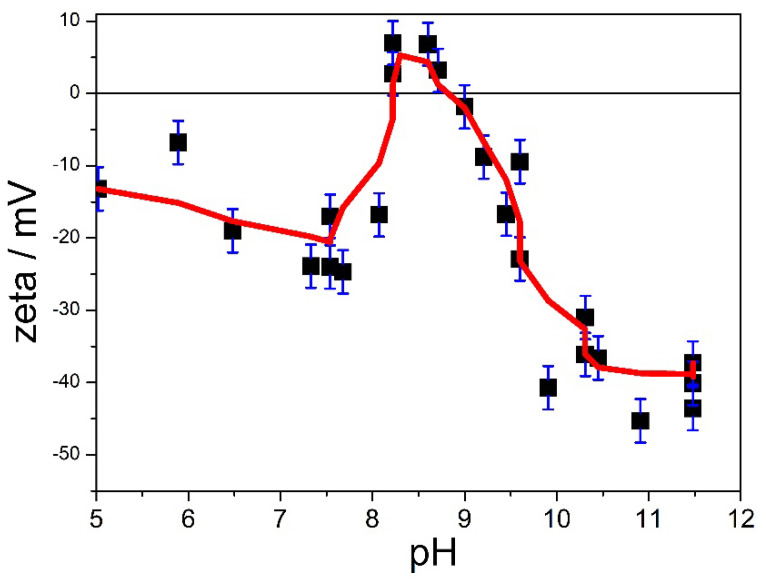
The electrokinetic curve of Z000 in 10^−3^ M NaCl.

The electrokinetic curve of Z000 in 10^−3^ M NaCl is presented in Figure 3. The curve is drawn to guide the eye; that is, it does not represent any theory. The error bars (±3 mV) represent a typical scatter of data points in electrokinetic measurements.

The segment between pH 8.3 and 11.5 is similar to the electrokinetic curves of metal oxides and many other materials; namely, the ζ potential decreases as the pH increases. Intersection of this segment with the pH axis indicates an isoelectric point (IEP) at pH 8.9, which is similar to the IEP of Zn oxide and hydroxide reported in many publications [19], but the course of the electrokinetic curve below pH 8.3 is very different from the electrokinetic curves of the Zn (hydr)oxide and (hydr)oxides of other metals. On the other hand, similar trends in electrokinetic curves, with maxima around pH 8, were reported for basic copper salts [14] and for basic zinc sulfate and chloride [20]. Such electrokinetic behavior of basic salts at low pH may be due to their conversion into other chemical compounds at low pH (selective leaching of OH^−^ ions), but it can also be due to conversion of basic salts into corresponding (hydr)oxides at high pH (selective leaching of sulfate ions).

### 2.2. Products of Ion Exchange

#### 2.2.1. XRD

**Figure 4:**
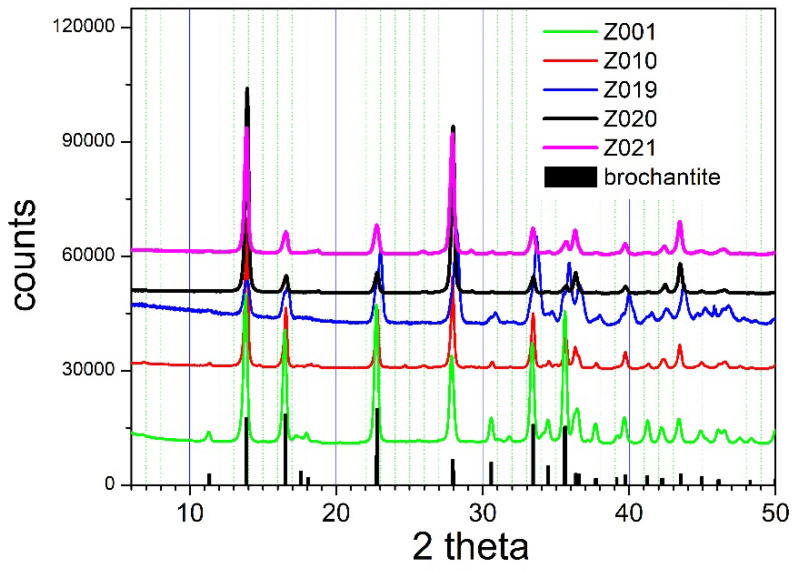
The XRD patterns of basic zinc sulfate completely converted into brochantite.

An extended analysis of the results reported in this section is presented in the Appendix A. According to reaction (5), about 1 g of CuSO_4_∙5 H_2_O is required to completely replace Zn with Cu in 0.5 g Zn_4_(OH)_6_SO_4_∙3.5 H_2_O. In a few cation-exchange experiments, the amount of CuSO_4_∙5 H_2_O was twice as high as the calculated stoichiometric amount (Table 1). In a few other cation-exchange experiments, the excess of CuSO_4_∙5 H_2_O was lower. The cation exchange in specimens Z001, Z010, Z019, Z020, and Z021 resulted in a complete conversion of Z000 into brochantite, as illustrated in Figure 4.

The XRD patterns of Z001, Z010, Z019, Z020, and Z021 show peaks characteristic for brochantite, and they do not show the peaks characteristic for Z000 (Figure 1) or for any other Zn compound. While the double excess of Cu with respect to the stoichiometric amount (reaction (5)) was used in Z001 and Z010, the excess of Cu in Z019, Z020, and Z021 was only 20, 40, and 60% with respect to the stoichiometric amount. This result indicates that a large excess of Cu is not essential for complete conversion. The amounts of Z001, Z010, Z019, Z020, and Z021 obtained by ion exchange were lower than the mass of the starting material (Z000), but they were greater by 5–10% than the stoichiometric amounts of brochantite (reaction (5)). This result may be due to limited control over the hydration level of reagents, but it can also be due to adsorption/coprecipitation of Zn on brochantite. The time of equilibration was 6 or 7 days for Z001, Z019, Z020, and Z021 but only 1 day for Z010. This result indicates that a 1-day equilibration may be sufficient to completely convert Z000 into brochantite at 40 °C. As discussed in the Introduction, isomorphic substitution of Zn by Cu in zinc basic sulfates is only possible up to a certain Cu level, and further cation exchange results in the crystallization of a new phase. Brochantite (monoclinic) differs from zinc basic sulfates in both structure (namuwite is hexagonal, and osakaite and lahnsteinite are triclinic) and hydration level (brochantite is anhydrous, and zinc basic sulfates are hydrated), so the ion exchange occurs due to dissolution–precipitation. It cannot be fully excluded that the initial stage of the ion exchange may occur via a topotactic substitution.

**Figure 5:**
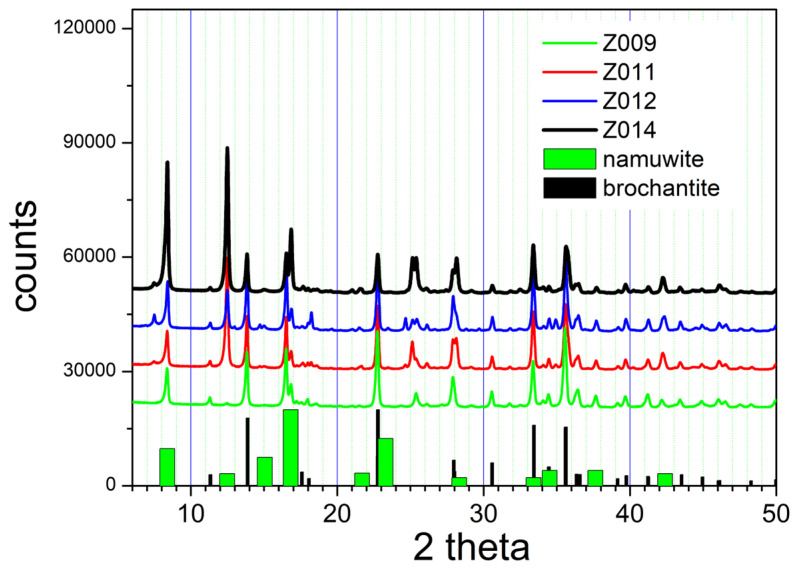
The XRD patterns of basic zinc sulfate partially converted into brochantite.

The cation exchange in specimens Z009, Z011, Z012, and Z014 resulted in a partial conversion of Z000 into brochantite, as illustrated in Figure 5.

The XRD patterns of Z009, Z011, Z012, and Z014 show peaks characteristic for brochantite, and they also show peaks characteristic for namuwite (4 hydrate), which is a component of Z000 (Figure 1). The presence of 3-hydrate in Z009, Z011, Z012, and Z014, which is also a component of Z000, is less obvious. The peaks at about 25.4° observed in Z009, Z011, Z012, and Z014 (and also in Z000) may be due to the 3-hydrate. In contrast, a peak at about 10° characteristic for 3-hydrate and observed in Z000 is absent in the XRD patterns of Z009, Z011, Z012, and Z014. The nearly stoichiometric amount of CuSO_4_∙5 H_2_O in Z009 (in contrast to the excess of CuSO_4_∙5 H_2_O in Z001, Z010, Z019, Z020, and Z021) is probably the reason for the incomplete conversion into brochantite (insufficient amount of Cu). The elevated temperature (50 or 60 °C) in Z011, Z012, and Z014 (in contrast with room temperature or 40 °C in Z001, Z010, Z019, Z020, and Z021) is probably the reason for the incomplete conversion into brochantite. Apparently, the equilibrium of reaction (5) is shifted to the right at low temperatures: That is, the reaction is exothermic. The masses of Z009, Z011, Z012, and Z014 were nearly equal to the masses of the starting material (Z000), and this confirms incomplete conversion (the stoichiometric amount of brochantite obtained in reaction (5) is lower than the mass of the starting material).

**Figure 6:**
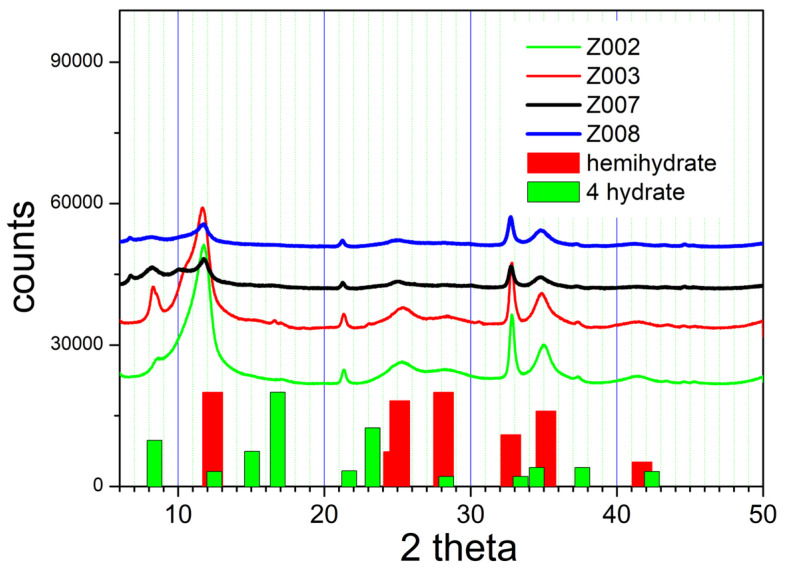
The XRD patterns of basic zinc sulfate after aging with Co(II) and Ni(II) sulfates.

The results of the attempted conversion of Z000 into basic sulfates of other transition metals are presented in Figure 6. Z003 and Z007, the products obtained with Co(II), were pink, and Z002 and Z008, the products obtained with Ni(II), were light green, but the only peaks found in their XRD patterns can be attributed to the different hydrates of basic zinc sulfate.

Moreover, the XRD patterns obtained with Co and Ni are very similar, and this suggests that, for example, Z007 (obtained in the presence of Co) and Z008 (obtained in the presence of Ni) represent the same product. In other words, equilibration of basic zinc sulfate with Co(II) and Ni(II) sulfates, followed by washing and drying, affects the hydration level of basic zinc sulfate, but it does not induce the transformation of basic zinc sulfate into another crystalline compound. This result is not very surprising for the following reasons:

- Lack of evidence for basic sulfates of Co and Ni in the literature.
- The Jahn–Teller effect is dependent on the number of d-electrons in divalent cations.
- The difference in ionic radii between Zn and Cu on the one hand and Co and Ni on the other.

The signal-to-noise ratio is poor in the XRD patterns presented in Figure 6 compared with Figure 1 and Figure 4, and 5, but several XRD patterns of basic zinc sulfates presented in the papers discussed in the Introduction also represent products with weak crystallinity.

**Figure 7:**
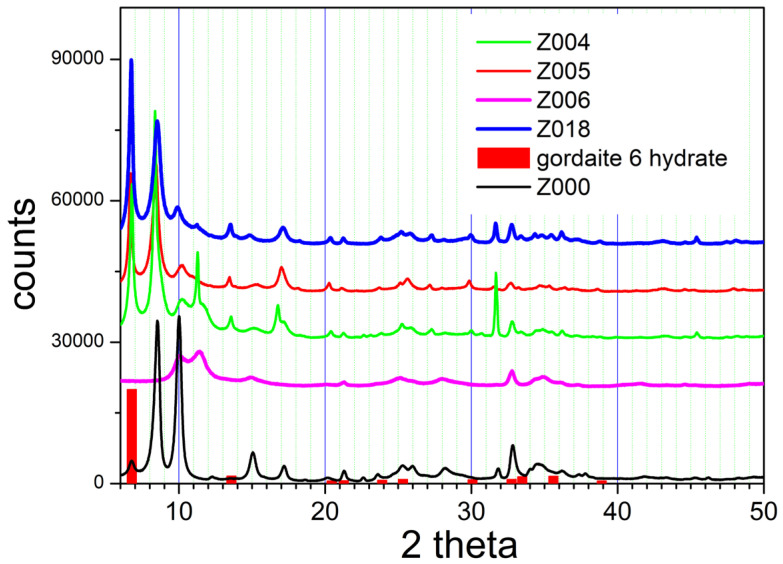
The XRD patterns of basic zinc sulfate after aging with alkali halides.

Figure 7 presents the XRD patterns of basic zinc sulfate after aging with alkali halides. The XRD patterns obtained upon aging with NaCl (Z004 and Z018) and NaBr (Z005) are all similar, but they are very different from the XRD pattern obtained upon aging with KI (Z006). The XRD patterns of Z004, Z018, and Z005 show several peaks that were also observed in the original material (Z000), which can be attributed to the different hydrates of Zn_4_(OH)_6_SO_4_. Additionally, they show a major peak at 6.7°, which can be interpreted as the main peak of gordaite (6 hydrate) and of its Br-analog. Unfortunately, the other peaks in the XRD pattern of gordaite reported by Delcheva et al. are not significant enough (or they match the peaks of namuwite) to confirm the presence or absence of gordaite in our materials. Our result is in line with the conversion of basic zinc sulfates into gordaite reported by Stanimirova et al. [10,15]. The gordaite peak is not found in the XRD pattern of Z006. This pattern indicates a weakly crystalline powder with most peaks similar to those in the original material (Z000) and an additional peak at 11.4°, which is similar to the peaks shown in Figure 6 and attributed to hemihydrate. The XRD patterns of materials obtained by ion exchange with potassium nitrate, acetate, and hydrogen phthalate are very similar to the XRD pattern of Z006. The similarity of the XRD patterns of the powders obtained by ion exchange with different salts confirms that Z002-008 and Z015-018 do not contain the ions of the salts used in the ion exchange (Table 1). This is emphasized again that none of the materials obtained by ion exchange has an XRD pattern identical to or even similar to the XRD pattern of the original material.

#### 2.2.2. BET

The BET specific surface areas of the materials obtained by ion exchange are presented in Table 1. The brochantites obtained by ion exchange at room temperature or at 40 °C with a substantial excess of CuSO_4_ (complete exchange, Figure 4) have BET specific surface areas higher by a factor of 2 than the original material. The brochantites obtained by ion exchange at 50 or 60 °C or with an insufficient excess of CuSO_4_ (partial exchange, Figure 5) have BET specific surface areas higher than the original material, but they are not as high as in pure brochantite (complete ion exchange).

#### 2.2.3. Electrokinetic Potential

**Figure 8:**
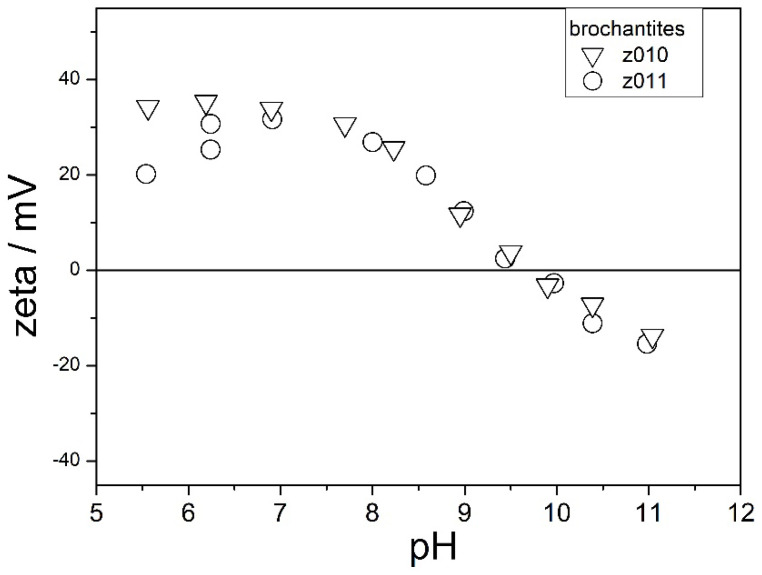
The electrokinetic curves of Z010 and Z011.

The electrokinetic curves of two brochantites are shown in Figure 8. The ion exchange was complete in Z010, but it was partially completed in Z011 (Figure 4 and Figure 5).

These electrokinetic curves have similar shapes, and such a shape is also typical for metal oxides; namely, the electrokinetic potential decreases with pH. These materials exhibit their IEPs at pH 9.5, which is similar to the IEPs of brochantite [14] and of CuO [19] reported in the literature.

**Figure 9:**
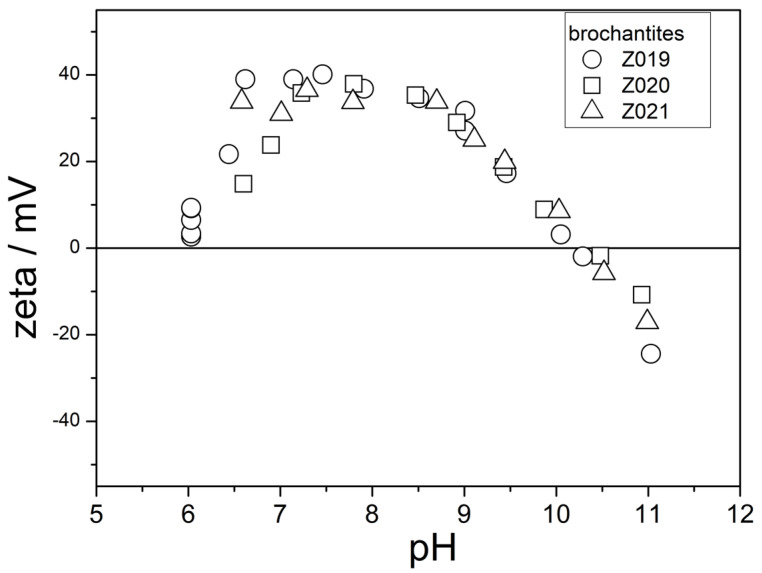
The electrokinetic curves of Z019, Z020, and Z021.

The electrokinetic curves of three other brochantites are shown in Figure 9. The ion exchange was complete in these materials (Figure 4).

These materials show their IEPs at pH 10, which is similar to the IEPs of brochantite [14] and of CuO [19] reported in the literature. However, the electrokinetic curves of Z019 and of Z020 show a clear maximum at a pH of about 8. Such a maximum cannot be confirmed for Z021, because data points for pH < 6.5 are not available for this material. In this respect, the electrokinetic curves in Figure 9 are different from those of metal oxides, for which the electrokinetic potential decreases with pH over the entire pH-range. On the other hand, the electrokinetic curves of Z019 and Z020 have similar shapes to the electrokinetic curve in Figure 1 and to the electrokinetic curves of basic salts presented in [14,20].

**Figure 10:**
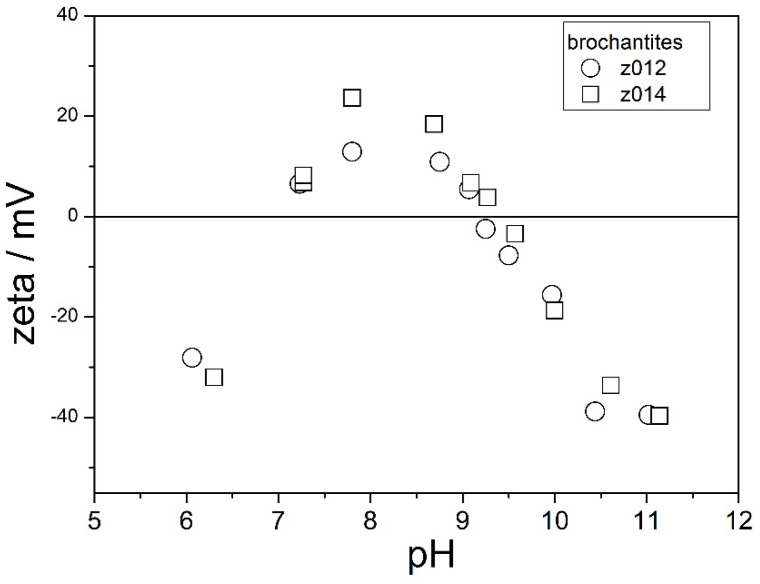
The electrokinetic curves of Z012 and Z014.

The electrokinetic curves of two brochantites are shown in Figure 10. The ion exchange was partially completed in these specimens (Figure 5).

The electrokinetic behavior of these materials is similar to the electrokinetic behavior of Z000 (the original basic zinc sulfate) and very different from the electrokinetic behavior shown in Figure 8 and Figure 9:

- The IEP is at pH 9.
- ζ potentials at pH about 6 are clearly negative.
- ζ potentials at pH about 11 are more negative (−40 mV) than those shown in Figure 8 and Figure 9 (−20 mV).

In other words, in spite of the presence of brochantite peaks in the XRD patterns of Z012 and Z014, their electrokinetic behavior (Figure 10) and their specific surface area (Table 1) make them more similar to the original Z000 than to the specimens completely converted into brochantite (Figure 8 and Figure 9). Similar trends in electrokinetic curves as those shown in Figure 10 (negative ζ potentials at sufficiently low pH) were observed in materials obtained by aging basic zinc sulfate with CoSO_4_ and NiSO_4_. These results support a hypothesis stating that the surface chemistry of these materials is dominated by basic zinc sulfate, even though clear brochantite peaks were found in Z012 and Z014 (Figure 5).

The choice of the reactor (plastic vs. glass) might have also influenced the course of the electrokinetic curves. It is well known that even trace amounts of silicate released from glass can adsorb on metal oxides like alumina and hematite and induce a more negative surface charge, thus resulting in a shift in the IEP to low pH. This is why contact with glassware is usually avoided in experiments involving ζ potential measurements, and plasticware is used instead. On the other hand, the composition of laboratory glass is designed to minimize its solubility in water. Contact with glass was avoided in the present study during the preparation of the original basic zinc sulfate and in most ion-exchange experiments. A shift in the IEP of brochantite from pH 10 (Figure 9) to 9.5 (Figure 8) and to 9 (Figure 10) may be due to the contact of Z010 and Z011 (Figure 8) and of Z012 and Z014 (Figure 10) with a glass reactor during the ion exchange, while the ion exchange in Z019, Z020, and Z021 (Figure 9) was conducted in plastic reactors. The presence of a maximum in ζ potential at pH 8 in several specimens and the absence of such a maximum in several other specimens might have also depended on the reactor in which the ion exchange was performed (plastic vs. glass); namely, the ion-exchange process resulting in Z019 and Z020 (Figure 9, maximum) was performed in plastic, while the ion exchange resulting in Z010 and Z011 (Figure 8, no maximum) was performed in glass. Traces of silicates might have prevented the selective leaching of OH^−^ ions from brochantite at low pH. On the other hand, the ion exchange leading to Z012 and Z014 (Figure 10, maximum) was also performed in glass, but basic zinc sulfate was only partially converted into brochantite in these specimens. Apparently, traces of silicate (released from a glass reactor) support a positive electrokinetic potential in the acidic range for materials fully converted into brochantite, but not for materials that are only partially converted into brochantite.

## 3. Materials and Methods

Most procedures were adopted from our previous work [14], and more details can be found there.

### 3.1. Reagents

The reagent-grade chemicals were from POCh, Lublin, Poland.

### 3.2. Preparation of Original Particles

The reaction

4 ZnSO_4_ + 6 NaOH → Zn_4_(OH)_6_SO_4_ + 3 Na_2_SO_4_ (4)

was used to obtain basic zinc sulfate. A dispersion of 71.8 g of ZnSO_4_ monohydrate in 200 mL of water (rather than a clear solution) was quickly poured into a fresh solution containing 24 g of NaOH in 200 mL of water under vigorous stirring. Stirring was continued for 1 day at room temperature. The final pH of the supernatant was 7.19, and the theoretical yield of anhydrous Zn_4_(OH)_6_SO_4_ was 55.9 g. The precipitate was washed with water as long as the supernatant produced a precipitate with Ba(NO_3_)_2_. Consecutive supernatants had a pH of about 7. The purified precipitate was dried at 60 °C in plastic for 1 day and at 95 °C in glass for 1 day, and the mass of the dried powder was 66.15 and 51.09 g, respectively. The powder was stored in an air-tight container but w/o control over the temperature or humidity level.

### 3.3. Ion Exchange

The replacement of Zn with Cu, described as

Zn_4_(OH)_6_SO_4_ + 4 CuSO_4_ → Cu_4_(OH)_6_SO_4_ + 4 ZnSO_4_ (5)

was the most studied reaction. On top of reaction (5), the exchange of other ions was attempted. The ion-exchange experiments were carried out using basic zinc sulfate from a single lot in air-tight plastic containers. A few experiments were performed in glass containers (marked as such in Table 1). Typically, about 0.5 g of basic zinc sulfate was shaken with 10 mL of solution containing about 1–2 g of water-soluble salt for 1 week. Then, the solid was separated by centrifugation and washed with water. The solids were dried at 105 °C and then stored in air-tight containers but w/o control over the temperature or humidity level. The conditions of the ion exchange are summarized in Table 1.

Z013 is missing in Table 1, because the experiment failed. The specimens without remarks in Table 1 underwent ion exchange under standard conditions: in a plastic container and for 7 days at room temperature. In a few specimens, the temperature was elevated to 40, 50, or 60 °C. In several specimens, the time of exchange was reduced to 1, 3, 5, or 6 d.

### 3.4. Characterization of Particles

As mentioned above, no special efforts were undertaken to control the temperature and humidity levels when the specimens were stored. XRD was used to confirm the conversion of basic zinc sulfate into another salt (Table 1). Empyrean from PANalytical (Malvern, UK) with Cu Kα radiation was used to collect the XRD patterns. Gemini V from Micromeritics (Norcross, GA, USA) was used to measure the adsorption of nitrogen at 77 K, and BET specific surface areas were calculated from the adsorption isotherms. The powders were dried at 130 °C prior to the adsorption measurement. This might have affected the hydration level of the powders, but the specimens are evacuated in the course of the measurement, and condensation of water during adsorption measurements must be avoided. The electrophoretic mobility was measured using a Malvern Zetasizer (Malvern, UK) at 25 °C in fresh dispersions of all specimens in 0.001 M NaCl at pH 6–11. The zeta potential was calculated using the Smoluchowski equation. The fresh original specimen (Z0), before rehydration in air, was also characterized by TGA (Netsch STA 449 F3 Jupiter, Selb, Germany) in an air flow, with the temperature raised at 10 K/min.

## 4. Conclusions

Aging of zinc hydroxy sulfate with an excess of CuSO_4_ solution leads to the complete or partial conversion of solid particles into brochantite, and the result depends on the excess of CuSO_4_, aging times, and temperatures. This ion-exchange behavior is unique for Cu; that is, analogous experiments with other transition metals (Co and Ni) do not result in the crystalline basic sulfates of these metals.

## Figures and Tables

**Table 1:** The specimens obtained by ion exchange.

Code	Basic Zinc Sulfate/g	Salt	Salt/g	Remarks	SSA m^2^/g
z000		none		Original basic zinc sulfate	3.96
z001	0.496	CuSO_4_·5 H_2_O	2.0016		9.6
z002	0.501	NiSO_4_·7 H_2_O	2.0034		6.44
z003	0.502	CoSO_4_·7 H_2_O	1.9956		4.49
z004	0.502	NaCl	2.0033		3.34
z005	0.507	NaBr	2.0039		4.57
z006	0.503	KI	2.0025		8.12
z007	0.478	CoSO_4_·7 H_2_O	1.4203	6 d	4.4
z008	0.478	NiSO_4_·7 H_2_O	1.4205	6 d	5.43
z009	0.478	CuSO_4_·5 H_2_O	1.0106	6 d	5.76
z010	0.501	CuSO_4_·5 H_2_O	2.0005	1 d, 40 °C, glass	9.52
z011	0.501	CuSO_4_·5 H_2_O	1.9999	1 d, 50 °C, glass	5.64
z012	0.501	CuSO_4_·5 H_2_O	2.0009	1 d, 60 °C	6.35
z014	0.502	CuSO_4_·5 H_2_O	2.0002	1 d, 50 °C	4.33
z015	0.501	KNO_3_	2.0018		6.6
z016	0.5	CH_3_COOK	2.0032		7.73
z017	0.502	KHC_8_H_4_O_4_	2.0036		3.07
z018	0.501	NaCl	2.0026		5.88
z019	0.502	CuSO_4_·5 H_2_O	1.2026	6 d	9.54
z020	0.501	CuSO_4_·5 H_2_O	1.4028	6 d	9.73
z021	0.484	CuSO_4_·5 H_2_O	1.6013	6 d	7.76
z022	0.502	CuSO_4_·5 H_2_O	2.0193	40 °C	6.21
z023	0.501	CuSO_4_·5 H_2_O	2.0093	5 d, 50 °C	6.01
z024	0.5	CuSO_4_·5 H_2_O	2.0133	60 °C	5.07
z025	0.507	NaCl	2.0011	glass	11.02
z026	0.5003	CuSO_4_·5 H_2_O	2.001	glass	7.54
z027	0.5004	CuSO_4_·5 H_2_O	1.0032	3 d, glass	8.72
z028	0.5022	CuSO_4_·5 H_2_O	1.0009	glass	11.54

**Figure A1:**
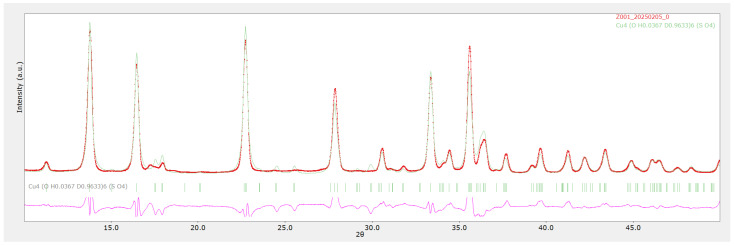
The XRD pattern of Z001. Red line: Z001; green line: brochantite; purple line: residues.

**Figure A2:**
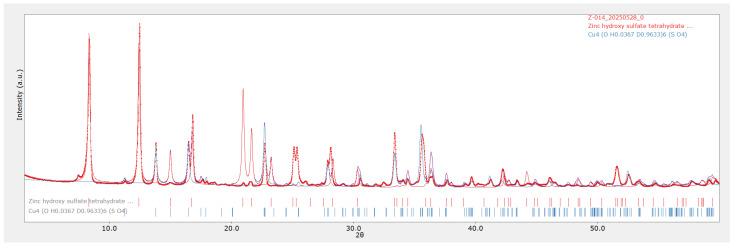
The XRD pattern of Z014. Thick red line: Z014; thin red line: Cu-substituted namuwite; blue line: brochantite.

**Figure A3:**
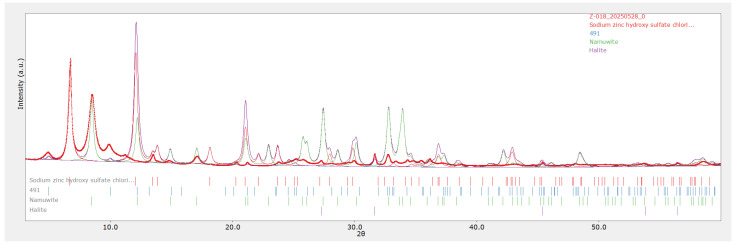
The XRD pattern of Z018. Thick red line: Z018; thin red line: gordaite; blue line: 2–2.25 hydrate of basic zinc sulfate (“new phase” discovered by Stanimirova et al.); green line: namuwite; purple line: halite.

**Figure A4:**
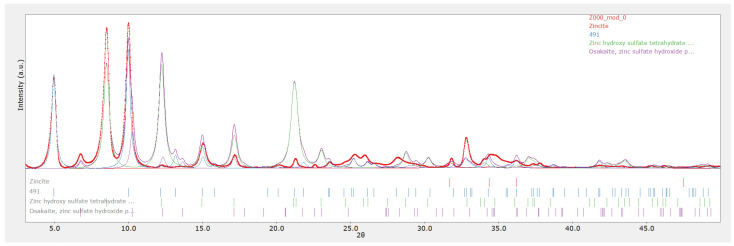
The XRD pattern of Z000. Thick red line: Z000; thin red line: zincite; blue line: 2–2.25 hydrate of basic zinc sulfate (“new phase” discovered by Stanimirova et al.); green line: namuwite; purple line: osakaite.

**Figure A5:**
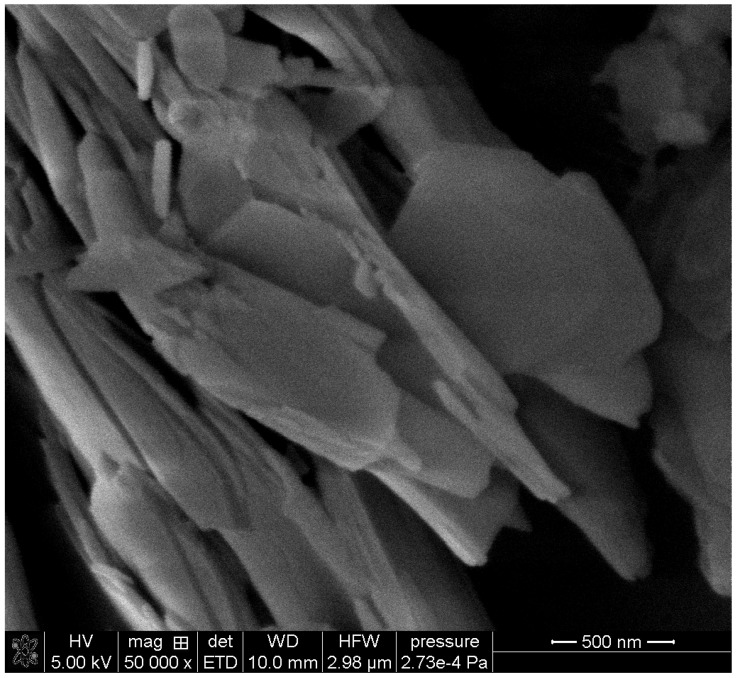
The SEM image of Z001.

## Data Availability

Data are available upon request from the corresponding author.

## Appendix A

Detailed analyses of selected XRD patterns are presented in this Appendix. Only a few representative XRD patterns are discussed in detail, but many other XRD patterns obtained in this study are very similar to those discussed in this Appendix. For example, all XRD patterns in Figure 4 are alike, and only one of them (Z001) underwent a detailed analysis. The specimens are ordered from the “easiest” (all peaks in the XRD pattern can be interpreted) to the most “difficult” (the XRD pattern does not unequivocally confirm the existence of crystalline phase(s)).

### Appendix A.1. Z001

The XRD pattern of Z001 (basic zinc sulfate after a complete cation exchange with CuSO_4_), as shown in Figure A1, represents pure brochantite without any trace of Zn. The crystallite size is 300 nm. A few reflections differ in their intensity from the prediction due to the orientation of crystallites, yet the effect of the preferential orientation of crystallites on the XRD pattern of Z001, as shown in Figure A1, is rather insignificant.

### Appendix A.2. Z014

The XRD pattern of Z014 (basic zinc sulfate after a partial cation exchange with CuSO_4_) shown in Figure A2 represents a mixture of brochantite (52% by volume and 73% by mass) with Cu-substituted namuwite (48% by volume and 27% by mass). The cell parameters in the Cu-substituted namuwite phase observed in Figure A2 (*a* = 0.815 nm, *c* = 1.049 nm) substantially differ from those reported for Zn-only namuwite (*a* = 0.833 nm, *c* = 1.054 nm), and the difference in the a-parameter reflects a partial substitution of Zn cations with Cu cations in the namuwite phase. The ionic radii of Cu and Zn are almost equal, but the aforementioned Jahn–Teller effect in Cu cations results in a distortion of the octahedra in the brucite-like layer and in their denser packing as compared with Zn cations. The d^9^ electronic configuration of Cu^2+^ in its octahedral complexes is the most-well-known example of the Jahn–Teller effect. On the other hand, the c-parameter represents about four water molecules per one (Zn,Cu)(OH)_6_SO_4_ unit, and it excludes the presence of higher (osakaite, *c* = 1.1) or lower (lahnsteinite, *c* = 0.93–0.97) hydrates. A minor difference in the c-parameter with respect to Zn-only namuwite in our sample may be due to a lower degree of hydration caused by the partial substitution of Zn cations with Cu cations in Z014.

The detailed analysis of the XRD pattern supports the aforementioned hypothesis stating that the initial stage of the cation exchange (ultimately resulting in conversion of basic zinc sulfate into brochantite) involves the isomorphic substitution of Zn with Cu in namuwite.

The XRD pattern of Z014 shown in Figure A2 is strongly affected by the preferential orientation of crystallites. Basic zinc sulfate occurs as platelets (cf. Appendix B), which are aligned parallel to the surface of the sample holder during the preparation of a specimen for XRD measurements. This results in enhanced 001 reflexes (8.44° in Figure A2) and weak reflexes of higher orders as compared with the random orientation of crystallites. For example, the reflection at about 15° (predicted for randomly oriented namuwite) is almost absent in the XRD pattern of Z014.

### Appendix A.3. Z018

Unlike in Appendix A.1 and Appendix A.2, where the entire XRD pattern could be fully explained in terms of one or two phases, the XRD pattern of Z018 (basic zinc sulfate after an attempted anion exchange with NaCl) involves more phases, and even then, the explanation is not satisfactory (cf. Figure A3). The XRD pattern of Z018 has a strong peak at 8.44° and a d-spacing of 1.047–1.05 nm, which corresponds to the basal (001) reflection in namuwite. On the other hand, a peak at about 12.1° predicted for namuwite is absent in the XRD pattern of Z018. This effect may be due to the preferential orientation of namuwite platelets, as discussed above for Z014.

The peak at 8.44° can also be interpreted as the basal (001) reflection of gordaite (d-spacing of 1.054–1.059 nm). Gordaite shows a preferential orientation of platelets similar to that of namuwite. This results in the absence of a peak at about 12.1° and of a few other peaks that might have occurred with a random orientation of the platelets. Minor signals at 27.38° and 31.7° are probably due to the presence of halite (NaCl). NaCl may be present in Z018 due to an insufficient washing procedure, but it could also have formed as a result of the breakdown of gordaite upon drying.

The Rietveld analysis and half-quantitative analysis resulted in the following composition of Z018: 70.7% by volume (37.4% by mass) of a phase described as sodium zinc hydroxy sulfate chloride (gordaite) and related materials, including the 2–2.25 hydrate of basic zinc sulfate (“new phase” discovered by Stanimirova et al.) and a material similar to namuwite; 23.8% by volume (57.8% by mass) of pure namuwite; and 4.7% by volume (3.9% by mass) of halite.

### Appendix A.4. Z000

Paradoxically, the interpretation of the XRD pattern of the initial material (Z000, Figure A4) was more demanding than the interpretation of the XRD patterns of most products of its ion exchange (Appendix A.1, Appendix A.2, Appendix A.3). As discussed above for Z014 and Z018, the preferential orientation of the platelets of basic Zn sulfates results in the absence of peaks in the XRD pattern of Z000 (for example, at about 12°) that are observed when platelets of basic zinc sulfates are randomly oriented. The material consists exclusively of Zn, S, O, and H; that is, Na, Cl, NH_4_, and C are absent. The size of the crystallites is within the range of 100–250 nm. Z000 consists of 65–75% of the 2–2.25 hydrate of basic zinc sulfate (“new phase” discovered by Stanimirova et al.), 10–15% osakaite, and 10–15% namuwite. Traces of the trihydrate of basic zinc sulfate (<5%) and ZnO (<2%) cannot be excluded.

### Appendix A.5. Z003

The attempts to interpret the XRD pattern of Z003 (basic zinc sulfate after attempted cation exchange with CoSO_4_) resulted in complete failure. A few peaks match those in Z000.

## Appendix B

The SEM image of Z000 is presented in this Appendix.

The SEM image presented in Figure A5 confirms that Z001 occurs in the form of hexagonal platelets, which are about 20 nm thick and about 500 nm in diameter. Similar images of basic zinc sulfates were obtained by others. The alignment of the platelets shown in Figure A5 explains the preferential orientation of crystallites in the XRD measurements and a very strong 001 peak in the XRD patterns, as discussed above.
